# Are Metals Emitted from Electronic Cigarettes a Reason for Health Concern? A Risk-Assessment Analysis of Currently Available Literature

**DOI:** 10.3390/ijerph120505215

**Published:** 2015-05-15

**Authors:** Konstantinos E. Farsalinos, Vassilis Voudris, Konstantinos Poulas

**Affiliations:** 1 Department of Cardiology, Onassis Cardiac Surgery Center, Sygrou 356, Kallithea 17674, Greece; E-Mail: vvoudris@otenet.gr; 2 Department of Pharmacy, University of Patras, Rio 26500, Greece; E-Mail: kpoulas@upatras.gr

**Keywords:** electronic cigarettes, smoking, metals, aerosol, risk assessment, inhalation

## Abstract

Background: Studies have found that metals are emitted to the electronic cigarette (EC) aerosol. However, the potential health impact of exposure to such metals has not been adequately defined. The purpose of this study was to perform a risk assessment analysis, evaluating the exposure of electronic cigarette (EC) users to metal emissions based on findings from the published literature. Methods: Two studies were found in the literature, measuring metals emitted to the aerosol from 13 EC products. We estimated that users take on average 600 EC puffs per day, but we evaluated the daily exposure from 1200 puffs. Estimates of exposure were compared with the chronic Permissible Daily Exposure (PDE) from inhalational medications defined by the U.S. Pharmacopeia (cadmium, chromium, copper, lead and nickel), the Minimal Risk Level (MRL) defined by the Agency for Toxic Substances and Disease Registry (manganese) and the Recommended Exposure Limit (REL) defined by the National Institute of Occupational Safety and Health (aluminum, barium, iron, tin, titanium, zinc and zirconium). Results: The average daily exposure from 13 EC products was 2.6 to 387 times lower than the safety cut-off point of PDEs, 325 times lower than the safety limit of MRL and 665 to 77,514 times lower than the safety cut-off point of RELs. Only one of the 13 products was found to result in exposure 10% higher than PDE for one metal (cadmium) at the extreme daily use of 1200 puffs. Significant differences in emissions between products were observed. Conclusions: Based on currently available data, overall exposure to metals from EC use is not expected to be of significant health concern for smokers switching to EC use, but is an unnecessary source of exposure for never-smokers. Metal analysis should be expanded to more products and exposure can be further reduced through improvements in product quality and appropriate choice of materials.

## 1. Introduction

Electronic cigarette (EC) use (vaping) is considered much safer than smoking [1,2,3], as ECs do not release combustion chemicals responsible for the health risks of smoking. EC devices are mainly composed of a battery part and an atomizer. The atomizer consists of a chamber usually composed of metals, plastics and/or glass, where the liquid is stored, and an atomizer head consisting of a wick and metal coil which is responsible for the heat delivery to the liquid that is subsequently evaporated. Due to this structure, some metal compounds may be delivered to the aerosol. Studies have reported the presence of metals in EC aerosol, in some cases at levels higher than in tobacco cigarette smoke [4,5]. However, no risk assessment was performed to evaluate the harmful potential from exposure to these metals. The purpose of this study was to examine the potential health risk associated with daily exposure to metals from EC use by comparing the highest levels found in the literature with maximum acceptable exposure limits defined by regulatory authorities.

## 2. Methods

### 2.1. Studies Evaluating Metals in ECs

The PubMed electronic database was searched, using the keywords “metals” and “electronic cigarette” and “e-cigarette”. Nine studies were found, with two of them evaluating the emissions of heavy metals in the aerosol [4,5] and another two evaluating environmental exposure [6,7]. The analysis herein focused on the former two studies, since higher levels of heavy metals are found in the aerosol collected from the EC compared to measuring environmental emissions. Inductively Coupled Plasma-Mass Spectrometry (ICP-MS) was used by Goniewicz *et al.* to measure heavy metals emissions to the EC aerosol of 12 products [4]. The levels found were compared with emissions from pharmaceutical nicotine inhalers. The following metals were analyzed: arsenic, barium, cadmium, chromium, cobalt, copper, lead, manganese, nickel, rubidium, strontium and zinc. Only three metals were detected in the EC aerosol (cadmium, lead and nickel). Williams *et al.* evaluated the presence of several metals in the aerosol emitted from one cartomizer and reported the results in amount per 10 puffs [5]. The following metals were found: aluminum, barium, chromium, copper, iron, lead, manganese, nickel, strontium, tin, titanium, zinc and zirconium. The authors did not clarify whether the reported values included subtraction of background levels found in room air, although they mentioned obtaining room samples. They also did not report metal concentrations in room air. Our analysis was based on the levels of metals reported in both studies and are presented separately for each product and as an average from all products. The samples tested by Goniewicz *et al.* [4] were named as EC01 to EC12, using the same terminology as the original study. For convenience, the sample tested by Williams *et al.* [5] was named EC13. Goniewicz *et al.* [4] reported the metal levels of blank samples (collection of air from the environment). In this analysis, the findings in the EC aerosol were corrected by subtracting the blank sample values from the EC aerosol values. No correction was made for the EC13 sample since Williams *et al.* [5] did not report the levels found in the environment. 

### 2.2. Exposure Limits

In order to assess the toxicity potential of the estimated daily exposure of these elements, we used the Permissible Daily Exposure (PDE) from inhalational medications, defined by the United States Pharmacopeia (USP) [8]. The PDEs refer to chronic exposure in adults of 50 kg body weight. For the metals not included in the USP list, we used Minimal Risk Level (MRL), defined by the Agency for Toxic Substances and Disease Registry (ATSDR) [9], or the Recommended Exposure Limit (REL), defined by the National Institute of Occupational Safety and Health (NIOSH) [10]. MRLs were developed in order to derive substance specific health guidance levels for non-neoplastic endpoints. An MRL is an estimate of the daily (24-h) human exposure to a hazardous substance that is likely to be without appreciable risk of adverse non-cancer health effects over a specified duration of exposure. ATSDR uses the No Observed Adverse Effect Level/Uncertainty Factor (NOAEL/UF) approach to derive MRLs for hazardous substances. They are set below those levels that, based on current evidence, might cause adverse health effects in the people most sensitive to such substance-induced effects. The MRLs for chronic inhalation exposure were used in the analysis herein. RELs were used when no data on PDEs and MRLs were available. RELs represent a time-weighted average concentration in the air for up to a 10-h workday during a 40-h workweek.

### 2.3. Assessing Daily Exposure from EC Use and Safety Limits

To evaluate daily exposure from EC use, the average daily number of puffs should be calculated. A study evaluating EC use topography defined an average consumption of 5 mg per puff, using a clearomizer [11]. Considering an average daily consumption of 3 g [12], we assumed an average use of 600 puffs per day. An uncertainty factor of 2, addressing inter-individual variability, was used; thus, the total daily exposure from 1200 EC puffs was estimated. The levels of metals found by Williams *et al.* [5] (reported per 10 puffs) were multiplied by 120 to obtain the level of daily exposure, while the levels found in the Goniewicz study [4] (reported per 150 puffs) were multiplied by 8, after subtracting background environmental levels. For MRLs and RELs, the average daily intake should be measured based on the amount of air inhaled over a period of 24 h for MRLs and 10 h for RELs. According to EPA, adults with moderate activity have an inhalation rate of 1.7 m^3^/h [13], leading to a 10-h and 24-h air volume of 17 m^3^ and 40.8 m^3^ respectively. Other organizations, such as the European Medicines Agency, use an average daily inhaled air volume of 20 m^3^/day in similar analyses [14]. We used the latter, and calculated the 10-h volume of air inhaled at 8.3 m^3^. Therefore, MRLs were multiplied with 20 and RELs were multiplied by 8.3, to calculate the acceptable daily exposure. In a secondary analysis, we compared the levels of daily exposure using a resting inhalation volume of 4.5 m^3^ per 10 h and 10.8 m^3^ for 24 h, based on an average resting respiratory rate for an adult of 15 breaths per minute and a tidal volume of 0.5 L [15,16]. MRLs were multiplied with 10.8 and RELs were multiplied by 4.5, to calculate the daily exposure. This is much stricter than usual assessments, since the calculated acceptable daily exposure levels (MRLs, RELs) are underestimated due to the low inhalation volume assumed. No secondary analysis was performed for the metals which were compared with PDEs from inhalational medications, since these safety limits are not associated with the amount of air inhaled.

## 3. Results

The results of the primary analysis and the ratio between regulatory limits and EC use daily exposure, considering a daily inhalation volume of 20 m^3^ air and a 10-h volume of 8.3 m^3^, are presented in Table 1. Chronic inhalational PDEs for medications were found for five metals, MRL was found for one metal and RELs were found for seven metals. No safety limits have been established for strontium. Table 2 shows the secondary analysis, considering a resting daily inhalation volume of 10.8 m^3^ air and a 10-h volume of 4.5 m^3^.

Aluminum is the most abundant metal in the Earth’s crust. It is used for consumer products, mostly in packaging, but also in medications, food additives and cosmetics [17]. It is also present in the atmosphere, at levels from 0.005 to 0.18 μg/m^3^, although in urban and industrial areas the levels may be from 4.0 to 8.0 μg/m^3^. Respiratory effects, in particular impaired lung function and fibrosis, have been observed in workers exposed to aluminum dust or fumes, although this has not been consistently observed across studies, raising the possibility that these effects may be due to dust overload rather than a direct effect of aluminum in lung tissue [17]. Of note, such effects were observed with heavy exposure to aluminum dust, in potroom and foundry workers [18,19]. EC atomizers structure as well as heating coils (e.g., kanthal coil) may contain aluminum. These are probably the main sources for aluminum in EC aerosol, justifying that it was the second most abundant element found by Williams *et al.* [5]. There are no established inhalational PDE or MRL for aluminum, but NIOSH has established a REL of 5000 μg/m^3^, leading to 41,500 μg/day total intake (assuming 10 h per day exposure) [20]. Aluminum was measured in the EC aerosol of sample EC13 only, with the daily exposure being >800 times below the safety limit. 

Barium is a silvery-white metal used in the manufacturing of plastics, paints, ceramics and steel hardening. The concentration in ambient air is <0.05 μg/m^3^ [21]. The most common natural forms are barium sulfate and carbonate. In one study, a benign pneumoconiosis was observed in several workers exposed to barium sulfate [22]; two other studies did not find barium-related alterations in the respiratory tract of workers exposed to barium sulfate [23] or barium carbonate [21]. The study by Williams *et al.* [5] did not report the barium form found in EC aerosol. No inhalational PDE or MRL have been established for barium or barium salts, thus we used the REL of barium nitrate (500 μg/m^3^), which is a soluble barium form with the lowest REL value [24]. No barium was found in samples EC01–EC12, while the daily exposure from EC13 use was found to be almost 3000 times below the safety limit.

**Table 1:** Exposure to metals from electronic cigarette use in comparison with regulatory safety limits from the primary analysis (assuming 20 m^3^ and 8.3 m^3^ respiratory volumes in 24-h and 10-h respectively); all values are in μg.

Metals	EC01		EC02		EC03		EC04		EC05		EC06		EC07		EC08		EC09		EC10		EC11		EC12		EC13	Average
**Cadmium**																										
*per 1200 puffs*	1.2		1.04		1.04		0		0.16		1.6		0		0.48		0		1.2		0.08		0		NM	0.57
*PDE (USP)*	1.5		1.5		1.5		1.5		1.5		1.5		1.5		1.5		1.5		1.5		1.5		1.5		1.5	
*Ratio (PDE/EC)*	1.3		1.4		1.4		−		9.4		0.9		−		3.1		−		1.3		18.8		−		−	2.6
**Chromium ^a^**																										
*per 1200 puffs*	0		0		0		0		0		0		0		0		0		0		0		0		0.84	0.06
*PDE (USP)*	25		25		25		25		25		25		25		25		25		25		25		25		25	
*Ratio (PDE/EC)*	−		−		−		−		−		−		−		−		−		−		−		−		29.8	386.9
**Copper**																										
*per 1200 puffs*	0		0		0		0		0		0		0		0		0		0		0		0		24.36	1.87
*PDE (USP)*	70		70		70		70		70		70		70		70		70		70		70		70		70	
*Ratio (PDE/EC)*	−		−		−		−		−		−		−		−		−		−		−		−		2.9	37.4
**Lead**																										
*per 1200 puffs*	0.32		0.32		0.4		0.08		0.24		0.08		0.16		4.4		0.56		0.32		0.16		0.08		2.04	0.70
*PDE (USP)*	5		5		5		5		5		5		5		5		5		5		5		5		5	
*Ratio (PDE/EC)*	15.6		15.6		12.5		62.5		20.8		62.5		31.3		1.1		8.9		15.6		31.3		62.5		2.5	7.1
**Nickel**																										
*per 1200 puffs*	0.88		0.96		0.32		0		0		0		0.48		0.72		0.16		0		0		0		0.6	0.32
*PDE (USP)*	1.5		1.5		1.5		1.5		1.5		1.5		1.5		1.5		1.5		1.5		1.5		1.5		1.5	
*Ratio (PDE/EC)*	1.7		1.6		4.7		−		−		−		3.1		2.1		9.4		−		−		−		2.5	4.7
**Manganese**																										
*per 1200 puffs*	0		0		0		0		0		0		0		0		0		0		0		0		0.24	0.02
*MRL (ATSDR)*	6		6		6		6		6		6		6		6		6		6		6		6		6	
*Ratio (MRL/EC)*	−		−		−		−		−		−		−		−		−		−		−		−		25.0	325.0
**Aluminum**																										
*per 1200 puffs*	NM		NM		NM		NM		NM		NM		NM		NM		NM		NM		NM		NM		47.28	47.28
*REL (NIOSH)*	41,500		41,500		41,500		41,500		41,500		41,500		41,500		41,500		41,500		41,500		41,500		41,500		41,500	
*Ratio (REL/EC)*	−		−		−		−		−		−		−		−		−		−		−		−		877.7	877.7
**Barium ^b^**																										
*per 1200 puffs*	0	0		0		0		0		0		0		0		0		0		0		0		1.44		0.11
*REL (NIOSH)*	4150	4150		4150		4150		4150		4150		4150		4150		4150		4150		4150		4150		4150		
*Ratio (REL/EC)*	−	−		−		−		−		−		−		−		−		−		−		−		2881.9		37,465.3
**Iron**																										
*per 1200 puffs*	NM	NM		NM		NM		NM		NM		NM		NM		NM		NM		NM		NM		62.4		62.40
*REL (NIOSH)*	41,500	41,500		41,500		41,500		41,500		41,500		41,500		41,500		41,500		41,500		41,500		41,500		41,500		
*Ratio (REL/EC)*	−	−		−		−		−		−		−		−		−		−		−		−		665.1		665.1
**Tin**																										
*per 1200 puffs*	NM	NM		NM		NM		NM		NM		NM		NM		NM		NM		NM		NM		4.44		4.44
*REL (NIOSH)*	16,600	16,600		16,600		16,600		16,600		16,600		16,600		16,600		16,600		16,600		16,600		16,600		16,600		
*Ratio (REL/EC)*	−	−		−		−		−		−		−		−		−		−		−		−		3738.7		3738.7
**Titanium ^c^**																										
*per 1200 puffs*	NM	NM		NM		NM		NM		NM		NM		NM		NM		NM		NM		NM		0.24		0.24
*REL (NIOSH)*	2490	2490		2490		2490		2490		2490		2490		2490		2490		2490		2490		2490		2490		
*Ratio (REL/EC)*	−	−		−		−		−		−		−		−		−		−		−		−		10,375.0		10,375.0
**Zinc**																										
*per 1200 puffs*	0	0		0		0		0		0		0		0		0		0		0		0		6.96		0.54
*REL (NIOSH)*	41,500	41,500		41,500		41,500		41,500		41,500		41,500		41,500		41,500		41,500		41,500		41,500		41,500		
*Ratio (REL/EC)*	−	−		−		−		−		−		−		−		−		−		−		−		5962.6		77,514.4
**Zirconium**																										
*per 1200 puffs*	NM	NM		NM		NM		NM		NM		NM		NM		NM		NM		NM		NM		0.84		0.84
*REL (NIOSH)*	41,500	41,500		41,500		41,500		41,500		41,500		41,500		41,500		41,500		41,500		41,500		41,500		41,500		
*Ratio (REL/EC)*	−	−		−		−		−		−		−		−		−		−		−		−		49,404.8		49,404.8

Abbreviations: EC, electronic cigarette; PDE, Permissible Daily Exposure; USP, United States Pharmacopeia; MRL, Minimal Risk Level; ATSDR, Agency for Toxic; Substances and Disease Registry; REL, Recommended Exposure Limit; NIOSH, National Institute of Occupational Safety and Health; NM, not measured; **^a^** Values for soluble forms of chromium III; **^b^** Values for barium nitrate, which has the lowest acceptable exposure limits among any barium salt; **^c^** Values for titanium dioxide.

Cadmium is a metal used in batteries, pigments, coatings and plastics. It exists in air as oxide, sulfate and chloride particles, with 5%–50% absorbed to the body through inhalation [25]. It is present in the soil and is highly absorbed by the tobacco plants, with subsequent exposure to smokers. Studies examining the toxicity of cadmium in workers have identified the respiratory tract and the kidney as sensitive targets of toxicity. Impaired lung function was reported in several studies [26,27], while other studies have not found any significant alterations [28]. According to the International Agency of Research on Cancer (IARC), cadmium is a known human carcinogen (Group 1) [29]. The average (EC01–EC12) levels of exposure from 1200 EC puffs were found to be 2.6 times lower compared to chronic PDE from licensed inhalational medications. Only one sample (EC06) was found to result in daily exposure 10% higher than PDE, while in four samples (EC04, EC07, EC09 and EC12) the levels were similar to background environmental air levels. 

Chromium is used for the manufacturing of several metal alloys such as stainless steel [30]. In its trivalent form (chromium III), it is an essential nutrient required for normal energy metabolism. Very low amounts are present in ambient air of urban areas (up to 30 ng/m^3^), but it is emitted from cigarette smoke, resulting in 10–400 times higher levels in indoor air. Respiratory exposure to the hexavalent form (chromium VI) elevates the risk of lung cancer, and the IARC officially classifies it as a known human carcinogen (group 1) [31]. Since it is an unstable form, it is highly unlikely that chromium VI is emitted to the EC aerosol. The most likely source of chromium in e-cigarettes is chromium-plated materials used in the atomizer structure and heating coils (both kanthal and nichrome contain chromium). The risk assessment analysis was performed by evaluating the chronic PDE levels from inhalational medications levels; EC daily exposure was found to be 30 times lower compared to PDE.

Copper, a reddish metal, is an essential element for all living organisms, including humans and plants [32]. The concentration of copper in the air is up to 200 ng/m^3^. It is a common metal used in the manufacturing of EC atomizers. No copper was found in samples EC01–EC12. The daily exposure from 1200 puffs of EC13 was approximately 30 times lower compared to the inhalational PDE from licensed medications.

Iron is an essential human nutrient with a variety of physiological roles including those associated with haemoglobin, myoglobin, ferritin and Fe-containing enzymes. It was the most abundant metal found in the study by Williams *et al.* [5], obviously because it is commonly used in the manufacturing of atomizers as well as in the heating coils. No inhalational PDE or MRL has been defined. NIOSH has defined a REL of 5000 μg/m^3^ for iron oxide [33]; assuming that iron oxide was the emitted compound, daily exposure from EC use (EC13) was >600 times lower compared to the safety limit. Iron was not measured in samples EC01–EC12.

Lead is a hazardous heavy metal, classified as a probable human carcinogen (group 2A) by the IARC [34]. It occurs naturally in the environment, but most of the high levels found result from human activities [35]. Lead was found in all tested products. Its origin in the study by Williams *et al.* [5] is unclear, considering that they did not find any lead in soldering joints (consistent with the ban on the use of lead in soldering material). All tested EC products emitted lead to the aerosol. The daily exposure from EC use was 7 times (average from all products) lower compared to the inhalational PDE levels in licensed medications. Large inter-product differences were observed; the amount of lead emitted varied by 50-fold between EC08 (4.4 μg/1200 puffs) and EC04, EC06 and EC12 (0.08 μg/1200 puffs).

Manganese is an essential nutrient, with several enzyme systems interacting with or depending on manganese for their catalytic or regulatory function [36]. It is important for the formation of healthy cartilage and bone, it aids in the maintenance of mitochondria and the production of glucose and it plays a key role in wound-healing. The mean concentration of manganese in ambient air in the United States is 0.02 μg/m^3^; however, ambient levels near industrial sources can range from 0.22 to 0.3 µg/m^3^. Exposure to high levels in the environment (2–22 mg/m^3^) may have disabling neurological effects in humans, leading to a disease called manganism. An MRL of 0.3 μg/m^3^ for chronic inhalation exposure has been established by ATSDR; daily exposure from EC13 was 25 times lower than the MRL. No manganese was found in products EC01–EC12.

Nickel is a hard, silvery-white metal, which has properties that make it very desirable for combining with other metals to form mixtures called alloys. Most nickel is used to make stainless steel [37]. Atmospheric nickel concentrations are higher in rural and urban air (concentration range: 5–35 ng/m^3^) than in remote areas (concentration range: 1–3 ng/m^3^) [38]. The most common harmful health effect of nickel in humans is an allergic reaction, with approximately 10%–20% of the population being sensitive to nickel [37]. Nickel compounds are classified as known human carcinogens by the IARC (Group 1). The most serious harmful health effects from exposure to nickel, such as chronic bronchitis, reduced lung function, and cancer of the lung and nasal sinus, have occurred in people who have breathed dust containing certain nickel compounds while working in nickel refineries or nickel-processing plants. However, the levels of nickel in these workplaces were much higher than usual (background) levels in the environment. Lung and nasal sinus cancers occurred in workers who were exposed to more than 10 mg/m^3^ of nickel, in the form of nickel compounds that were hard to dissolve (such as nickel subsulfide) [38]. A potential source of nickel in e-cigarettes is nichrome heating wire, as well as other parts of the atomizer which could be made from this material. Daily exposure from EC use (average of all products) was almost 5 times lower compared to the chronic inhalational PDE level. Six products did not emit any nickel to the aerosol, while for the rest the range of daily exposure was 1.6–9.4 times lower compared to PDE.

Strontium is usually found in nature in the form of minerals. Strontium compounds, such as strontium carbonate, are used in making ceramics and glass products, pyrotechnics, paint pigments, fluorescent lights, medicines, and other products [39]. The average amount of strontium that has been measured in air from different parts of the US is 20 ng/m^3^. There are no harmful effects of stable strontium in humans at the levels typically found in the environment. The only chemical form of stable strontium that is very harmful by inhalation is strontium chromate, but this is because of toxic chromium and not strontium itself. Ordinary strontium salts are not harmful when inhaled or placed on the skin. The radioactive form is classified as a known human carcinogen by IARC (Group 1), however, it seems unlikely that such a form was emitted from the ECs. No chronic inhalation PDE, MRL or REL have been defined for strontium. Although strontium was measured in all samples, only the aerosol of EC13 had detectable levels.

Tin is a soft, white, silvery metal that is insoluble in water. Inorganic tin compounds (tin combined with chlorine, sulfur, or oxygen) are found in toothpaste, perfumes, soaps, coloring agents, food additives, and dyes. Stannic oxide dust or fumes produce a benign form of pneumoconiosis in humans, known as stannosis [40]. Workers exhibiting this pulmonary condition had industrial exposures ranging from 15 to 20 years. No exposure levels were included in the case reports. No impairment of pulmonary function or systemic disease was observed. It has also been reported that x-rays of tin foundry workers confirmed more than 150 cases of stannosis by 1959 [41]. Tin in ECs is probably derived from soldering joints. Daily exposure from EC13 was >3000 times lower than the NIOSH-established REL of 2000 μg/m^3^ [42]. Tin was not measured in samples EC01–EC12.

Titanium is a biocompatible metal, used in medicine for bone fracture healing and joint replacement. Concerns about titanium relates only to titanium tetrachloride and titanium dioxide. The former is not found naturally in the air, is very unstable and enters the environment primarily as air emissions from facilities that make or use it in various chemical processes or as a result of spills [43]; therefore, it is extremely unlikely that it was emitted from EC use. Titanium dioxide is formed from oxidation of titanium. There is a concern about its carcinogenicity potential, with IARC classifying it as a possible human carcinogen (Group 2B) [44]. However, a recent review evaluated the mortality statistics at 11 European and four US titanium dioxide manufacturing plants and concluded that there was no suggestion of any carcinogenic effect associated with workplace exposure to titanium dioxide [45]. Although the study by Williams *et al.* [5] does not clarify which titanium compound was emitted from the ECs tested, our analysis assumed that titanium dioxide was emitted. NIOSH has established a REL of 2400 μg/m^3^ for fine and 300 μg/m^3^ for ultrafine particles [46]. Even when considering the worst-case scenario (ultrafine particles), daily exposure from EC13 was >10,000 times lower compared to the safety limit. Titanium was not measured in samples EC01–EC12.

Zinc is an essential nutrient, with zinc compounds commonly used by the pharmaceutical industry as ingredients in some common products, such as vitamin supplements, sun blocks, diaper rash ointments, deodorants, athlete's foot preparations, acne and poison ivy preparations, and antidandruff shampoos. [47]. Average levels of zinc in the air throughout the United States are usually less than 1 µg/m^3^, but range from 0.1 to 1.7 µg/m^3^ in areas near cities [47]. Inhaling large amounts of zinc (as zinc dust or fumes from smelting or welding) can cause a specific short-term disease called metal fume fever, which is generally reversible once exposure to zinc ceases. The NIOSH-established REL for zinc is 5000 μg/m^3^, thus, the daily exposure from EC13 was almost 6000 times lower than the safety limits. No zinc was found in samples EC01–EC12.

Zirconium is a soft grayish to gold-colored metal used in nuclear industry, corrosion resistant alloys, explosives, vacuum tube and iron and steel manufacturing. Additionally, it has some biomedical uses, such as dental implants, knee and hip joint replacements [48]. Daily exposure from EC13 was >49,000 times lower compared to the NIOSH-established REL of 5000 μg/m^3^ for zirconium [49]. Zirconium was not measured in samples EC01–EC12.

Table 2 shows the secondary analysis, considering a resting inhalation volume of 10.8 m^3^ in 24-h and 4.5 m^3^ in 10-h. This significantly underestimates the acceptable daily exposure levels from MRLs and RELs. Still, the daily exposure ranged from EC use ranged from >300 (for iron) to >40,000 (for zinc) lower than RELs and was 13.5 lower than MRL for manganese.

**Table 2:** Exposure to metals from electronic cigarette use in comparison with regulatory safety limits from the secondary analysis (assuming a resting 10.3 m^3^ and 4.5 m^3^ respiratory volumes in 24-h and 10-h respectively); all values are in μg; the metals analyzed based on United States Pharmacopeia Permissible Daily Exposures (PDEs) are not presented because PDEs are defined as level of exposure per day, irrespective of the respiratory volume (see Table 1).

Metals	EC01		EC02		EC03		EC04		EC05		EC06		EC07		EC08		EC09		EC10		EC11		EC12		EC13	Average
**Manganese**																										
*per 1200 puffs*	0		0		0		0		0		0		0		0		0		0		0		0		0.24	0.02
*MRL (ATSDR)*	3.24		3.24		3.24		3.24		3.24		3.24		3.24		3.24		3.24		3.24		3.24		3.24		3.24	
*Ratio (MRL/EC)*	−		−		−		−		−		−		−		−		−		−		−		−		13.5	175.5
**Aluminum**																										
*per 1200 puffs*	NM		NM		NM		NM		NM		NM		NM		NM		NM		NM		NM		NM		47.28	47.28
*REL (NIOSH)*	22,500		22,500		22,500		22,500		22,500		22,500		22,500		22,500		22,500		22,500		22,500		22,500		22,500	
*Ratio (REL/EC)*	−		−		−		−		−		−		−		−		−		−		−		−		475.9	475.9
**Barium ^a^**																										
*per 1200 puffs*	0		0		0		0		0		0		0		0		0		0		0		0		1.44	0.11
*REL (NIOSH)*	2250		2250		2250		2250		2250		2250		2250		2250		2250		2250		2250		2250		2250	
*Ratio (REL/EC)*	−		−		−		−		−		−		−		−		−		−		−		−		1562.5	20312.5
**Iron**																										
*per 1200 puffs*	NM		NM		NM		NM		NM		NM		NM		NM		NM		NM		NM		NM		62.4	62.40
*REL (NIOSH)*	22,500		22,500		22,500		22,500		22,500		22,500		22,500		22,500		22,500		22,500		22,500		22,500		22,500	
*Ratio (REL/EC)*	−		−		−		−		−		−		−		−		−		−		−		−		360.6	360.6
**Tin**																										
*per 1200 puffs*	NM		NM		NM		NM		NM		NM		NM		NM		NM		NM		NM		NM		4.44	4.44
*REL (NIOSH)*	9000		9000		9000		9000		9000		9000		9000		9000		9000		9000		9000		9000		9000	
*Ratio (REL/EC)*	−		−		−		−		−		−		−		−		−		−		−		−		2027.0	2027.0
**Titanium ^b^**																										
*per 1200 puffs*	NM		NM		NM		NM		NM		NM		NM		NM		NM		NM		NM		NM		0.24	0.24
*REL (NIOSH)*	1350		1350		1350		1350		1350		1350		1350		1350		1350		1350		1350		1350		1350	
*Ratio (REL/EC)*	−		−		−		−		−		−		−		−		−		−		−		−		5625.0	5625.0
**Zinc**																										
*per 1200 puffs*	0	0		0		0		0		0		0		0		0		0		0		0		6.96		0.54
*REL (NIOSH)*	22,500	22,500		22,500		22,500		22,500		22,500		22,500		22,500		22,500		22,500		22,500		22,500		22,500		
*Ratio (REL/EC)*	−	−		−		−		−		−		−		−		−		−		−		−		3232.8		42,025.9
**Zirconium**																										
*per 1200 puffs*	NM	NM		NM		NM		NM		NM		NM		NM		NM		NM		NM		NM		0.84		0.84
*REL (NIOSH)*	22,500	22,500		22,500		22,500		22,500		22,500		22,500		22,500		22,500		22,500		22,500		22,500		22,500		
*Ratio (REL/EC)*	−	−		−		−		−		−		−		−		−		−		−		−		26,785.7		26,785.7

Abbreviations: EC, electronic cigarette; MRL, Minimal Risk Level; ATSDR, Agency for Toxic Substances and Disease Registry; REL, Recommended Exposure Limit; NIOSH, National Institute of Occupational Safety and Health; NM, not measured; **^a^** Values for barium nitrate, which has the lowest acceptable exposure limits among any barium salt; **^b^** Values for titanium dioxide.

## 4. Discussion

In this study the hazardous potential of metals emitted to EC aerosol was evaluated, by using regulatory standards of acceptable exposure. The results show that the levels of daily exposure from EC use are significantly lower compared to acceptable exposure from inhalational medications and by orders of magnitude lower than the regulatory limits for daily occupational exposure. It is important to emphasize that such analysis was performed by using worst case scenarios: the daily exposure from ECs was overestimated (doubling the average number of daily puffs), while in the secondary analysis we additionally assumed that a person breaths with the resting respiratory rate and tidal volume over the whole day (underestimating the level of acceptable daily exposure calculated from MRLs and RELs). Still, the levels of metals exposure from EC use were of minimal apparent health concern. Exposure to multiple metals is also observed in medicinal products such as nicotine inhalers [4]. However, it should be emphasized that there were significant differences in exposure between samples, meaning that it is possible to further reduce exposure to metals by improving production quality. Moreover, the e-cigarette aerosol is a complex mixture of several compounds other than meats. The analysis herein presents a risk assessment limited to metals exposure. Finally, this analysis does not take into account the exposure to metals in EC users from other sources (such as daily activities or occupational exposure). However, the regulatory limits are also set for specific situations (e.g., medications use for PDE and occupational setting for NIOSH) and do not exclude the possibility of additional exposure from other activities or in other settings.

The chronic PDE levels for inhalational medications were considered the most appropriate (and probably the most stringent) comparator to assess the impact of EC exposure. Similarly to medications, any potential hazard from EC use should be evaluated in the context of the benefit associated with reduction or complete cessation of tobacco cigarette use, since the targeted population is smokers. It is the balance between risks and benefits that determines whether a product should be recommended for use. It is reasonable to assume that the PDE levels defined by USP for inhalational medications are not absolutely safe, but still the benefits outweigh any potential risks. A similar principle applies to EC use when used as a substitute for smoking, while it should be emphasized that any exposure is unnecessary for never-smokers. Although in most cases the emissions from ECs resulted in metal exposure much lower (in many cases by orders of magnitude) than PDE levels, some ECs had cadmium and nickel exposure levels lower by only 30%–60% compared to PDE, while one product (EC06) was the only one with cadmium exposure levels higher (by 10%) than the PDE. This smaller difference could be associated with the stringency of the US Pharmacopeia safety limits, as well as with the heating coil of the atomizers which are a potential source of nickel.

MRLs defined by ATSDR are very conservative estimates, taking into account the NOAELs and dividing them by an UF associated with interspecies differences and inter-individual variability. Exposure to manganese from EC13 was 25 times lower compared to the established MRL, while no manganese was found in samples EC01–EC12. Even when we considered the unlikely scenario of 24-h respiration with the resting respiratory rate and tidal volume, the levels of daily exposure from EC12 were still 13.5 times lower than MRL. 

RELs defined by NIOSH are related to industrial hygiene and refer to maximum levels of compounds that the workers are exposed to for a limited proportion of the day (10-h per day, 40-h per week). Unlike PDEs and MRLs, RELs are probably higher than acceptable levels for exposure for the general population or sensitive population groups. However, in all cases, the levels of metals found in EC aerosol were lower by >2–4 orders of magnitude compared to RELs, even in the worst case scenario. This difference is higher than most UFs applied in risk assessment. It should be emphasized that we evaluated emissions to which the EC user is directly exposed; it is expected that environmental emissions are much lower and, thus, of no significant concern for the health of bystanders (second-hand exposure). 

The results of this risk assessment are not in agreement with concerns over adverse health effects of EC metal emissions expressed by Williams *et al.* [5]. In that study, a list of adverse health effects was presented, associated with chronic exposure to very large concentrations of metals while much lower levels were found in the study. This emphasizes the importance of performing a risk assessment, which will result in proper guidance to clinicians and reliable information to the targeted population group (smokers) about any relevant health concern.

Although the average level of metals exposure from the EC products tested do not justify serious health concerns, significant differences in emissions were observed between EC products, especially for metals of high concern such as cadmium, lead and nickel. For cadmium, one product (EC06) would result in higher than PDE exposure levels under the extreme EC use conditions we evaluated (1200 puffs per day). On the contrary, no cadmium was detected in the aerosol of three products (EC04, EC07 and EC12). A similar pattern was observed with lead. It was detected in all samples but differences up to 50-fold in daily exposure was found between products. Nickel was not emitted by six products while a 6-fold difference in emissions was observed between the other products. Some products resulted in higher exposure compared to the nicotine inhalator, although statistical significance was not observed for most of the products [4]. These findings indicate that there is a large margin for improvement, by implementing strict quality standards and choosing appropriate materials. EC manufactures should evaluate metal emissions in their products and should aim to avoid unnecessary exposure. 

This study evaluated the risk associated with exposure from 13 products, compared to the hundreds of products available in the market. Evaluation of all available products is probably non-feasible as a research project but could be implemented through regulatory standards, at least for the metals of major concern. Use of biocompatible materials that would resist corrosion when in contact with liquids and under the repeated heating and cooling cycles of EC use could result in reduced exposure to metals. More studies are needed to define which materials are more appropriate for EC atomizers. Moreover, the size of solid particles such as metals may affect the exposure outcome through enhanced penetration in tissues and organs. ECs emit particles in nanometer range; however, metals have not been characterized in terms of their size in EC aerosol. Although there are no regulatory limits specifically defined for the particle size of metal emissions, we assume that size has been considered when defining the safety limits since it is well known that nanoparticles are abundant in occupational settings [50,51,52]. Another issue is that health risk may be associated with the solubility of different metal particle forms [53]. For example, nickel is more toxic in insoluble form, mainly oxidic and sulfidic nickel species [54], compared to soluble forms [55]. Solubility may also affect the site of harm, with water soluble metals being able to penetrate the lung easier, reducing risk of lung disease but enhancing potential risk for disease in other organs [56]. No such specific assessment was performed in this analysis, due to the lack of data on the form of metals detected in EC aerosol. Moreover, the regulatory limits do not usually define separate levels of exposure depending on the metal form, probably because in occupational settings (metallurgy industry) there is an abundance of many forms of metals that the workers are exposed. The study by Williams *et al.* [5] evaluated an outdated cartomizer model. There is a continuous improvement in the design, properties and safety features of EC parts, with new-generation atomizers featuring pyrex glass and stainless steel structure substituting plastics and other metals (for example: http://www.kangeronline.com/products/aerotank-turbo-clearomizer and http://www.aspirecig.com/Nautilus-Tank-System-c74670086.html). Further studies should evaluate whether these advancements result in different metal emissions compared to those studied so far. Research should also focus on defining the exact source of metal emissions. For example, the most commonly used heating coils, kanthal and nichrome, may be an important source of nickel and chromium emitted to the aerosol. This, as well as the potential to reduce emissions by using alternative materials for heating coils, needs to be evaluated. Furthermore, both studies analyzed herein evaluated products used for the first time during the study sessions. Atomizers are used for several days before being discarded (or atomizer heads changed), thus it is important to identify whether there is any change in the stability and related metal emissions of the atomizer components (mainly the heating coil) after some days of use. In any case, some metal emissions are expected since the atomizers are essentially metallic structures, and this might be preferable to using materials such as plastics which could interact with liquid and results in the release of plasticizers. 

## 5. Conclusions 

In conclusion, the levels of metals emitted to the EC aerosol, as found in currently available literature, are unlikely to generate significant adverse health effects for smokers switching to EC use. However, discrepancies between products indicate that standards in product quality should be implemented in order further reduce unnecessary exposure to metals. 
